# Effect of Innovative Bio-Based Plastics on Early Growth of Higher Plants

**DOI:** 10.3390/polym15020438

**Published:** 2023-01-13

**Authors:** Ewa Liwarska-Bizukojc

**Affiliations:** Institute of Environmental Engineering and Building Installations, Lodz University of Technology, Al. Politechniki 6, 90-924 Lodz, Poland; ewa.liwarska-bizukojc@p.lodz.pl; Tel.: +48-42-631-35-22

**Keywords:** bio-based plastics, plant growth, seed germination, terrestrial ecosystem

## Abstract

Plastic particles are widespread in the environment including the terrestrial ecosystems. They may change the physicochemical properties of soil and subsequently affect plant growth. In recent decades, traditional, petroleum-derived plastics have been increasingly replaced by more environmentally friendly bio-based plastics. Due to the growing role of bio-based plastics it is necessary to thoroughly study their impact on the biotic part of ecosystems. This work aimed for the assessment of the effect of five innovative bio-based plastics of different chemical composition and application on the early growth of higher plants (sorghum, cress and mustard). Each bio-based plastic was tested individually. It was found that the early stages of growth of monocotyledonous plants were usually not affected by any of plastic materials studied. At the same time, the presence of some kinds of bio-based plastics contributed to the inhibition of root growth and stimulation of shoot growth of dicotyledonous plants. Two PLA-based plastics inhibited root growth of dicotyledonous plants more strongly than other plastic materials; however, the reduction of root length did not exceed 22% compared to the control runs. PBS-based plastic contributed to the stimulation of shoot growth of higher plants (sorghum, cress and mustard) at the concentrations from 0.02 to 0.095% *w/w*. In the case of cress shoots exposed to this plastic the hormetic effect was observed. *Lepidium sativum* turned out to be the most sensitive plant to the presence of bio-based plastic particles in the soil. Thus, it should be included in the assessment of the effect of bio-based plastics on plant growth.

## 1. Introduction

Soil plays many important ecological functions such as accumulation and filtration of water and nutrients, transformation of chemicals, biomass production and carbon storage [1]. The presence of chemical contaminants in soil reduces these functions and aggravates soil properties. Micro- and nanoplastic particles became one of the most common pollutants in the terrestrial ecosystems in the last decades [2]. It was demonstrated that they changed soil properties, including soil aggregation, bulk density and water holding capacity [3,4]. In order to evaluate soil quality and vitality, apart from the determination of chemical and physical indicators, the use of soil biota is recommended [5,6].

Plants are one of the most important functional groups of organisms in the terrestrial ecosystems [6]. They produce oxygen and food for other living creatures, as well as they are regulators of key ecosystem processes and services, e.g., carbon dynamics and sequestration, nutrient dynamics and soil structural stability [7]. Thus, various plant species are used as biological indicators in the soil ecotoxicity tests. With regard to the evaluation of the effects of microplastics on higher plants, the two following species have been frequently assessed: *Lepidium sativum* representing dicotyledonous plants [8,9,10] and *Triticum aestivum* being a monocotyledonous plant [11,12,13]. In addition, other plants such as *Daucus carota* [14] and *Allium fistulosum* [15] were used for testing the potential impacts of plastic particles on the organisms representing producers in the food chain. However, it should be emphasized that the number of studies concerning the assessment of the effects of plastic particles on higher plants that have been published so far is very limited.

It was found that microplastics usually did not affect the seed germination processes of either mono- or dicotyledonous plants [8,12,13]. This concerns both petroleum-derived and bio-based plastic particles. At the same time, plastic particles might act on the early growth of plants stimulating or inhibiting it [9,16]. Balestri et al. [9] observed that a significant number of seedlings exposed to leachates from high-density polyethylene (HDPE) or the bio-based biodegradable plastic Materbi^®^ showed developmental abnormalities or seedling growth reduction. Polylactide (PLA) and polyhydroxybutyrate (PHB) microparticles at the concentration 11.9% *w/w* contributed to the inhibition of root growth of *Sinapsis alba* and *Lepidium sativum* [16]. At the same time, Lozano et al. [14] reported about the increase in the growth of *Daucus carota* cultivated for 28 days in the soil containing from 0.1 to 0.4% (*w/w*) plastic microparticles made of polyester (PES), polyamide (PA), polypropylene (PP), low-density polyethylene (LDPE), poly(ethylene terephthalate) (PET), polyurethane (PU), polystyrene (PS) and polycarbonate (PC). Huerta Lwanga et al. [13] did not observe any effect of PLA on *Triticum aestivum* growth.

In this work, five newly synthesized bio-based plastics were tested with regard to their potential impact on seed germination and the early growth of plants. All bio-based plastics were obtained with the cooperation of the project Bio-plastic Europe (Horizon 2020, grant agreement no. 860407). It was hypothesized that the materials tested would not affect seed germination but they might inhibit or stimulate the early growth of roots and/or shoots. In order to verify these hypotheses, a series of early growth tests using three different higher plants as model organisms were carried out.

## 2. Materials and Methods

### 2.1. Bio-Based Plastics

Five innovative bio-based plastic materials subjected to study were received due to the realization of Bio-plastic Europe Project (Horizon 2020, grant agreement no. 860407). Three of them were provided by NaturePlast SAS (NP, Mondeville, France) and these were the following compounds: BPE-AMF-PLA (Bio-Plastic Europe—Agriculture Mulch Film—PolyLactic Acid), BPE-T-PHBV (Bio-Plastic Europe—Toys—Poly Hydroxy Butyrate Valerate), BPE-SP-PBS (Bio-Plastic Europe—soft Packaging—PolyButylene Succinate), while the other two, i.e., BPE-C-PLA (Bio-Plastic Europe—Rigid Packaging—PolyLactic Acid), BPE-RP-PLA (Bio-Plastic Europe—Rigid Packaging—PolyLactic Acid) were provided by Arctic Biomaterials OY Ltd. (ABI, Tampere, Finland). All bio-based plastics were supplied by the manufacturers in the form of microparticles. The abbreviations of tested bio-based plastics were the same as it was assumed in the project nomenclature. The characteristics of the plastic materials tested are shown in Table 1.

### 2.2. Methods of Evaluation of Phytotoxicity

Impacts of bio-based plastics on plants were assessed in agreement with ISO Standards 18763 [17] using the commercial toxicity bioassay—Phytotoxkit Solid Samples provided by Microbiotests (Ghent, Belgium). This assay enables for the determination of the number of germinated seeds and the growth of roots and shoots of selected higher plants exposed to the contaminated matrix (the reference soil containing plastic particles) compared with the controls (the reference soil only). Five following concentrations of plastic particles in the reference OECD soil were tested: 0.02, 0.095, 0.48, 2.38 and 11.9% *w/w*. The tests for each particle concentration were made in three replications for each plastic material and each plant, whereas the control tests were made in nine replications for each plant. The monocotyledonous plant *Sorghum saccharatum* (sorghum, series no. SOS041019) and two dicotyledonous plants *Lepidium sativum* (garden cress, series no. LES260820) and *Sinapis alba* (mustard, series no. SIA020719) were used as model organisms in these experiments. The appropriately prepared soil and ten seeds of one of the higher plants were placed in the special test plate dedicated to this assay. All these materials (the reference soil, seeds and test plates) were delivered by Microbiotests (Ghent, Belgium). Then, all test plates were incubated for 72 h at 25 ± 1 °C in the darkness in the acclimation chamber FITO 700 (Biogenet, Józefów, Poland). After incubation the number of germinated seeds was recorded for each test and control plate and germination index was calculated [16]. Additionally, a digital picture of each plate was made and then subjected to image analysis using the NIS ELEMENTS AR software (Nikon, Japan). As a result, the lengths of roots and shoots were measured. The composition of the OECD reference soil and more detailed description of the phytotoxicity assay used in this work are presented elsewhere [16].

### 2.3. Statistical Analysis

The results of measurements were subjected to statistical elaboration. It comprised basically the calculation of mean values, standard deviation and goodness of normal distribution. The latter was checked with the use of the Kolmogorov–Smirnov test. In addition, one-way analysis of variance (ANOVA) at statistical significance α = 0.05 was applied to evaluate whether the lengths of roots or shoots of plants exposed to one of the plastics tested were statistically equal or different than those that were not exposed to the plastics. As the null hypothesis it was assumed that they were equal. The statistical elaboration of results was performed with the use of MS Excel (Analysis ToolPak) software and OriginPro 9.0 (OriginLab, Northampton, MA, USA).

## 3. Results and Discussion

**Figure 1:**
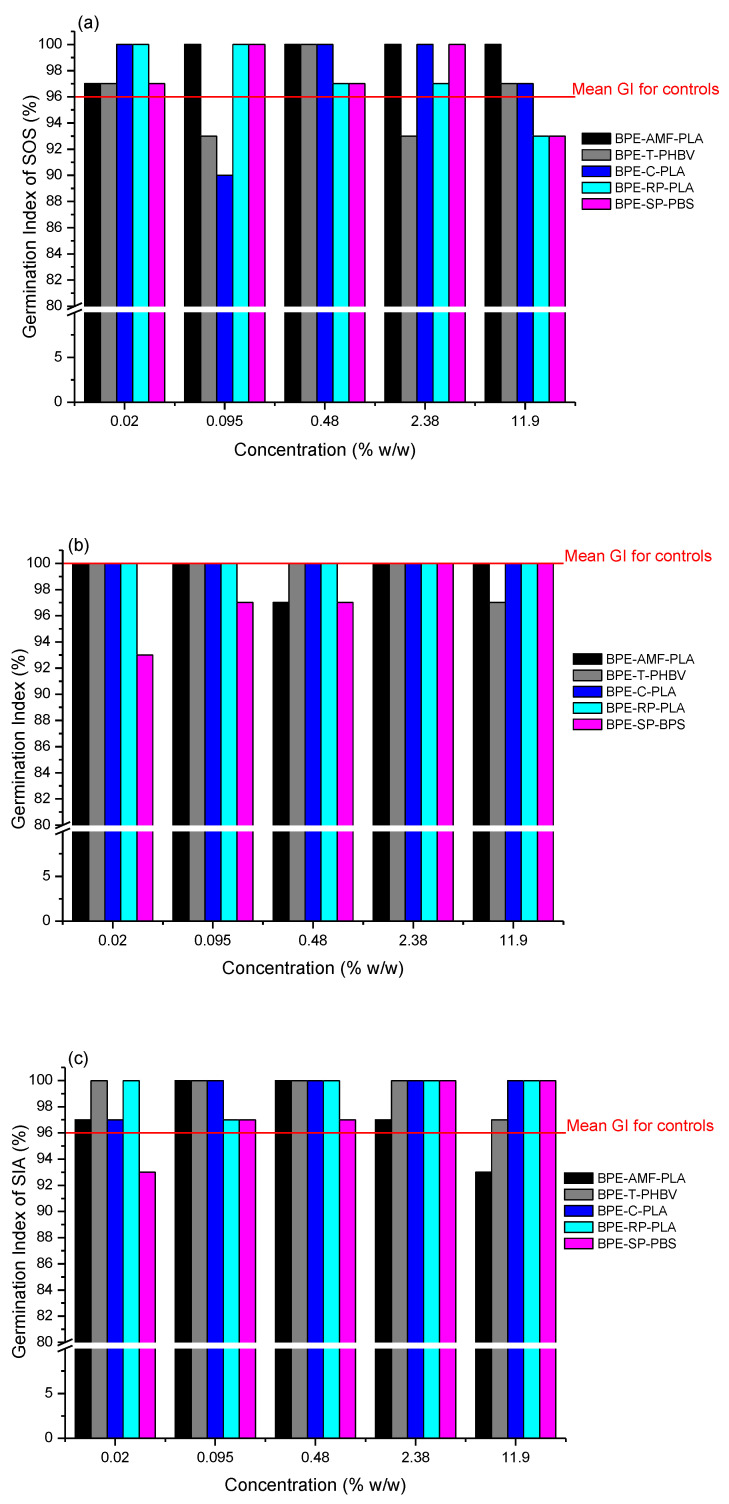
Effect of bio-based plastics on seed germination of higher plants: (**a**) *Sorghum saccharatum* (SOS); (**b**) *Lepidium sativum* (LES); (**c**) *Sinapsis alba* (SIA).

Seed germination is a key process in the seed plant life cycle that influences total biomass yield and quality [18,19]. This process depends on both intrinsic (e.g., seed dormancy and available food store) and extrinsic (e.g., temperature, light, relative humidity, chemicals) factors [19,20]. Thus, the presence of plastic particles in the soil may influence it. Bio-based plastics studied in this work did not hamper the seed germination processes of any of three higher plants used as model organisms. The values of GI in the tests with the addition of plastic particles were approximately at the same level as those determined in the control tests (Figure 1). This was observed for each plant, i.e., monocotyledonous *S. saccharatum* as well as dicotyledonous *L. sativum* and *S. alba*. The results of one-way ANOVA confirmed that there were not statistically significant differences between the germination efficiency in the tests with and without plastic particles in the soil. The *p*-values were in the range from 0.3319 to 0.9515 for sorghum, while for the cress and mustard they were from 0.05039 to 0.1245 and from 0.05004 to 0.9478, respectively. Consequently, they were less than or equal to the significance level α = 0.05. Other studies on this subject also showed that plastic microparticles did not affect the germination of wheat [12] or cress [9,16]. This proves that germination of seed plants is relatively uninfluenced by the soil composition [9].

The analysis of the effect of bio-based plastics on early growth of higher plants comprised both the development of roots and shoots. The results of measurements of plant roots and shoots varied widely, and they were difficult to use for interpretation in spite of being subjected to statistical elaboration. Depending on the compound tested and its concentration in the soil, inhibition and/or stimulation of root/shoot growth or no impact were found. A high degree of variability of results of phytotoxicity tests was also observed in other studies concerning petroleum-derived and/or bio-based plastics [11,14,21].

**Figure 2:**
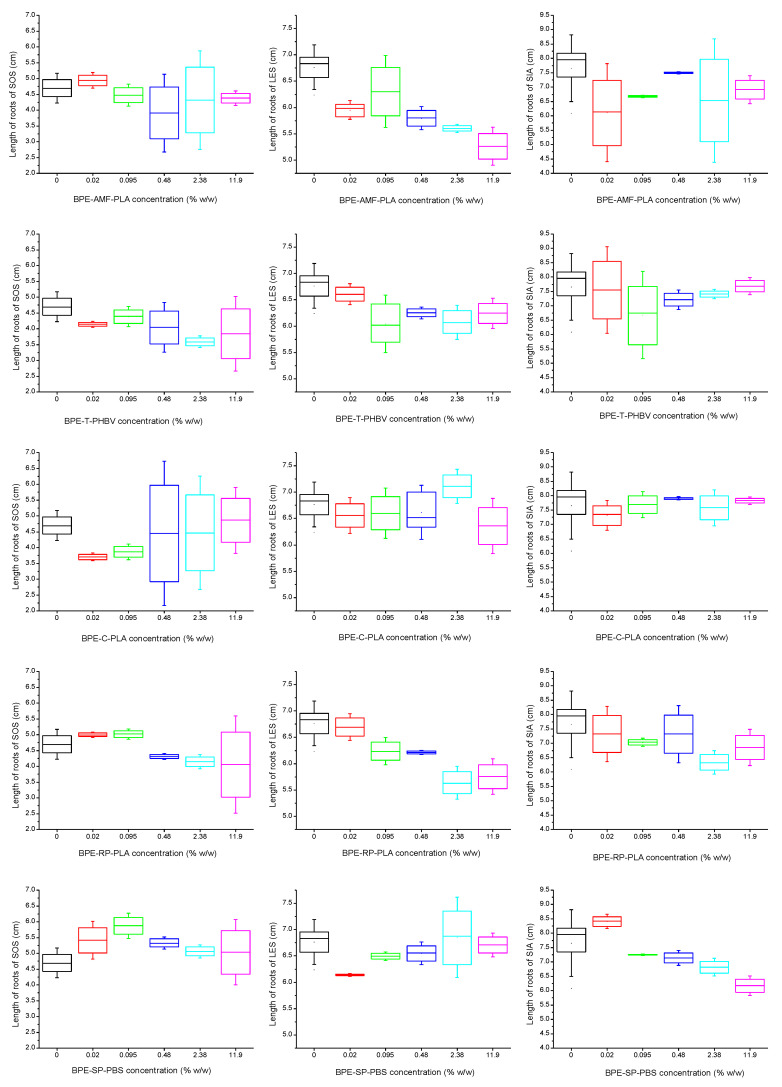
Effect of bio-based plastics on root growth of higher plants: *Sorghum saccharatum* (SOS), *Lepidium sativum* (LES) and *Sinapsis alba* (SIA).

Roots are regarded to be the first organ exposed to the impact of toxic compounds present in the soil. As a consequence, roots react to these stress conditions mainly by growth inhibition [22]. The changes of root length of each of higher plant used as bioindicators in this study are depicted in Figure 2. The growth of sorghum roots was not inhibited by any of bio-based plastic tested irrespective of its concentration in the soil. This was statistically confirmed with the help of one-way ANOVA (*p* > 0.05) (Table 2). Only in the case of BPE-SP-PBS, was the stimulation of root growth observed at the two lowest concentrations tested (*p* < 0.05) as seen in Figure 2, and Table 2. At the same time the cress root growth was inhibited by three out of five bio-based plastics tested, BPE-AMF-PLA, BPE-T-PHBV and BPE-RP-PLA. This was found for each concentration (BPE-AMF-PLA) or for four out of five (BPE-T-PHBV, BPE-RP-PLA) concentrations of bio-based plastic particles in the soil (Table 2). The presence of the other two materials (BPE-C-PLA, BPE-SP-PBS) in the soil did not affect the root growth of cress significantly (Figure 2, Table 2). With regard to mustard, the inhibition of root growth was revealed in the tests with BPE-AMF-PLA, BPE-RP-PLA and BPE-SP-PBS. However, this was not found in every case over the entire range of concentrations of plastic particles in the soil. In the case of BPE-AMF-PLA, this was found in three out of five concentrations of this material in the soil, while in the case of BPE-RP-PLA or BPE-SP-PBS, the inhibition of root growth was statistically confirmed at the two highest concentrations (Figure 2, Table 2).

The results of root length obtained for three plant species showed that dicotyledons were more sensitive to the presence of bio-based plastic particles in the soil than monocotyledons. At the same time, in the case of the stress factor induced by stimuli other than plastic particle soil contaminations (e.g., metals), the response of monocotyledons might be the same or stronger in comparison to dicotyledons [22,23]. Out of three plants used as bioindicators, cress appeared to be the most sensitive organism in the assessment of the effect of bio-based plastic materials on the early stages of root growth. Comparing to what extent each bio-based plastic affected root length, it was observed that two bio-based plastics (BPE-AMF-PLA and BPE-RP-PLA) acted more strongly on root growth than other plastic materials did. In particular, it affected the root growth of cress and mustard. The presence of BPE-AMF-PLA or BPE-RP-PLA in the soil contributed to the decrease of cress roots from 6.3 to 21.8% or from 0.6 to 16.4%, respectively. In the case of mustard roots, it was from 2.1 to 19.8% in the tests with BPE-AMF-PLA and from 4.3 to 17.4% in the tests with BPE-RP-PLA (Figure 2). The reduction of root length of any plant did not exceed 21.8% irrespective of the type of bio-based plastic added to the soil (Figure 2).

**Figure 3:**
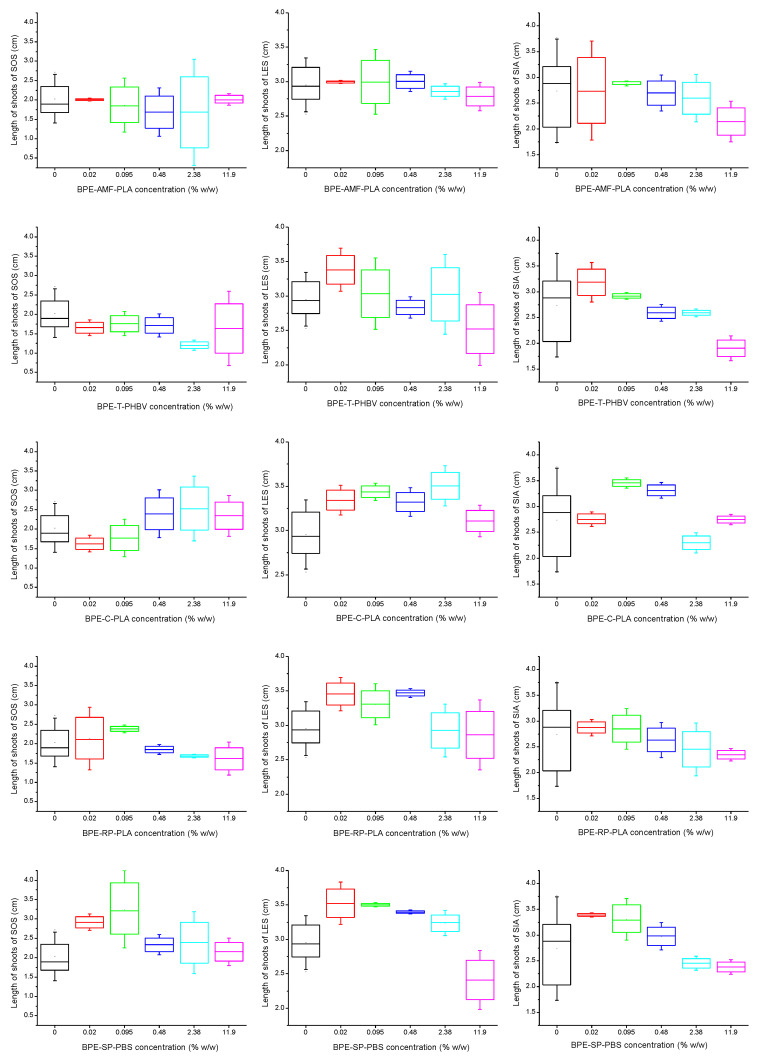
Effect of bio-based plastics on shoot growth of higher plants: *Sorghum saccharatum* (SOS), *Lepidium sativum* (LES) and *Sinapsis alba* (SIA).

Shoot growth, just like root growth, was affected by bio-based plastics in different ways depending mainly on the plant used as the indicator, the type of bio-based plastic and its concentration in the soil (Figure 3). In the case of shoot growth, the stimulation was more often observed than it was noted for roots (Table 2). The stimulatory effect of low doses of toxic substances on growth of plants or animals is a known phenomenon called as hormesis [24,25,26]. The growth of sorghum shoots was generally not inhibited by the presence of plastic material in the soil (Table 2). At the same time, BPE-SP-PBS contributed to the stimulation of growth of sorghum shoots at the lower concentrations 0.02–0.095% *w/w* of plastic particles in the soil (Figure 3, Table 2).

Shoot development of cress was the most often stimulated in the tests with the addition of the bio-based plastic particles to the soil (Figure 3, Table 2). This phenomenon was observed at almost all concentrations tested in the case of three out five bio-based plastics tested, namely, BPE-C-PLA, BPE-RP-PLA and BPE-SP-PBS.

The inhibition of cress shoot growth was found only at the highest concentrations of bio-based plastic particles in the soil. It concerned the tests with BPE-SP-PBS and BPE-T-PHBV. At the same time, the presence of BPE-AMF-PLA did not affect shoot growth of cress at all (Figure 3, Table 2). The impact of bio-based plastic particles on mustard shoot development was weaker than that with regard to cress. The inhibition was observed at the highest concentration (11.9% *w/w*) in the case of tests with BPE-AMF-PLA or BPE-T-PHBV, while the presence of BPE-SP-PBS or BPE-C-PLA stimulated the growth of mustard shoots at the concentrations 0.02–0.095% *w/w* or 0.095–0.48% *w/w*, respectively (Figure 3, Table 2).

As was the case with the roots, cress turned out to be the most sensitive model organism in the evaluation of the effect of bio-based plastic particles on shoot growth. It is in line with the previous results concerning PLA, PHB and polypropylene (PP), which indicated that *L. sativum* was the most sensitive plant in the tests with these three plastics [16]. Therefore, this plant should be used as one of bioindicators in the assessment of potential phytotoxicity of plastics, in particular bio-based plastic materials.

Comparing the effect of the individual plastics on shoot growth, it was easy to see that BPE-SP-PBS stimulated the growth of shoots of all higher plants tested, when it was present in the soil at concentrations from 0.02 to 0.095% *w/w*. In the case of cress at lower concentrations of plastic particles in the soil, the stimulation of cress shoot growth occurred, while at the highest concentration (11.9% *w/w*) their growth was inhibited (Figure 3, Table 2). This response of the biological parameter (shoot growth) in the presence of BPE-SP-PBS in the soil at various concentrations (stimulation at lower concentrations, inhibition at higher concentrations) corresponded with the description of the hormetic effect [25,26]. Thus, the hormetic effect of bio-based plastic BPE-SP-PBS on shoot growth of cress is very probable.

## 4. Conclusions

In this work, five bio-based plastics of different chemical composition and various potential applications were studied towards their impact on early growth of higher plants.

None of bio-based plastics even at relatively high concentration in the soil (11.9% *w/w*) interferes with the germination of plant seeds. This is the case of monocotyledonous and dicotyledonous plants.

The growth of roots of *S. saccharatum* (monocotyledonous plant) is not inhibited irrespective of the type of bio-based plastic and its concentration in the soil. At the same time, the growth of roots of dicotyledonous plants is inhibited by some kinds of bio-based plastics. The inhibition of the development of *L. sativum* roots is statistically confirmed for two PLA-based plastics and one PHBV-based plastic. In the case of roots of *S. alba* it is confirmed for the same two PLA-based plastics as for cress and additionally for PBS-based plastic.

Two PLA-based plastics, BPE-AMF-PLA and BPE-RP-PLA, inhibit root growth of dicotylodonous plants more strongly than other plastic materials. Nevertheless, the shortening of roots does not exceed 22% in comparison to the control runs in any case.

Shoot growth of the monocotyledonous plant is usually not affected by the presence of bio-based plastics in the soil. With regard to the dicotyledonous plants, in particular cress shoots, stimulation of growth often occurs. This is statistically confirmed for the growth of *L. sativum* shoots exposed to BPE-C-PLA, BPE-RP-PLA or BPE-SP-PBS. The presence of PBS-based plastic in the soil contributes to the stimulation of shoot growth of higher plants (sorghum, cress and mustard) at the concentrations from 0.02 to 0.095% *w/w*. In the case of cress shoots exposed to PBS-based plastic, the hormetic effect is observed.

This work confirms that *L. sativum* is the most sensitive plant out of three bioindicators used with regard to the presence of bio-based plastic particles in the soil. It is recommended to include *L. sativum* in the evaluation of the effect of bio-based plastics on the early growth of higher plants.

## Figures and Tables

**Table 1:** Data on the bio-based plastics tested (provided by the manufacturers).

Acronym of Bio-Based Plastic	APPLICATION	Desired Properties	Material Type	Density g cm^−3^	Size of Granules	Innovation	Material Details	Manufacturer
BPE-AMF-PLA	Mulch film	Bio-based and both recyclable and bio-degradable, degrades in controlled fashion	PLA-based	1.26	Length 3 mm; diameter 2.5 mm	Blending of PLA and polyhydroxy butyrate-hydroxyvalerate (PHBV) for controlled degradation, fertilizer added for controlled release	PLA blended with 15% polybutylene adipate terephthalate (PBAT) and <5% process additives, intended to be used for extrusion application	NaturePlast SAS (Mondeville, France)
BPE-T-PHBV	Toys	Recyclable, industrially compostable rheology, thermal stability, melt viscosity, resistance to hydrolysis, migration	PHBV-based	1.24	Length 3 mm; diameter 2.5 mm	Blending of PLA, and soft unsaturated PHAs, first mechanical, thermal characterization (smallest scale); in vitro (enzymatic) degradation	PHVBV blended with <15% additives (mostly impact modifier), intended to be used for injection application	NaturePlast SAS (Mondeville, France)
BPE-SP-PBS	Soft Packa-ging	Recyclable, industrially compostable rheology, thermal stability, melt viscosity, resistance to hydrolysis, barrier properties	PBS-based	1.26	Length 3 mm; diameter 2.5 mm	Improve processing and hydrolysis resistance, testing of recyclability, blending with PLA to increase mechanical properties (soft packaging to more rigid packaging)	PBS blended with <15% additives (mostly mineral filler), intended to be used for thermoforming or injection application	NaturePlast SAS (Mondeville, France)
BPE-C-PLA	Cutlery	Reusable cutlery with good mechanical properties and heat resistance	PLA-based	1.40	Length 3 mm; diameter 2.5 mm	Thermal stability, processing, resistance to hydrolysis, suitable for dishwasher cleaning, environmental degradation, ecotoxicology	PLA-based compound filled with 20% of degradable glass fiber	Arctic Biomaterials OY Ltd. (Tampere, Finland)
BPE-RP-PLA	Rigid packa-ging	Water and oxygen barrier, bio-based and bio- degradable	PLA-based	1.50	Length 3 mm; diameter 2.5 mm	Cold mold, fast cycle time, good heat resistance, food grade	PLA-based mineral filled compound (food grade) for injection molding and potentially sheets for thermoforming	Arctic Biomaterials OY Ltd. (Tampere, Finland)

**Table 2:** Results of one-way ANOVA for *S. saccharatum* (SOS), *L. sativum* (LES) and *S. alba* (SIA).

Tested compound	Exposed plant organ	*p*-values for SOS				
		Concentrations (% *w/w*)				
		0.02	0.095	0.48	2.38	11.9
BPE-AMF-PLA	roots	0.6231	0.5702	0.0678	0.3654	0.6693
	shoots	0.9034	0.6639	0.01781 (I)	0.3142	0.7238
BPE-T-PHBV	roots	0.1868	0.4567	0.6671	0.01371(I)	0.7165
	shoots	0.1638	0.4523	0.3511	0.00735(I)	0.9482
BPE-C-PLA	roots	0.02192(I)	0.06123	0.7624	0.2606	0.7531
	shoots	0.2482	0.5049	0.1488	0.07073	0.2089
BPE-RP-PLA	roots	0.5214	0.5082	0.3626	0.1977	0.1625
	shoots	0.6457	0.1868	0.6723	0.3267	0.4432
BPE-SP-PBS	roots	0.03223(S)	0.008538(S)	0.2049	0.4288	0.8969
	shoots	0.001491(S)	1.78·10^−5^(S)	0.2255	0.03400(S)	0.5318
Tested compound	Exposed plant organ	*p*-values for LES				
		Concentrations (% *w/w*)				
		0.02	0.095	0.48	2.38	11.9
BPE-AMF-PLA	roots	2.78·10^−5^(I)	0.0371(I)	3.72·10^−6^(I)	7.13·10^−8^(I)	7.21·10^−12^(I)
	shoots	0.639	0.716	0.378	0.389	0.136
BPE-T-PHBV	roots	0.4633	0.000132(I)	0.00855(I)	0.00195(I)	0.0471(I)
	shoots	0.000159(S)	0.477	0.277	0.570	0.000662 (I)
BPE-C-PLA	roots	0.386	0.499	0.253	0.0335 (S)	0.0507 (I)
	shoots	0.000834(S)	2.09·10^−5^(S)	0.00311(S)	7.19·10^−8^(S)	0.194
BPE-RP-PLA	roots	0.826	0.00488(I)	0.00329(I)	9.25·10^−8^(I)	1.46·10^−8^(I)
	shoots	3.90·10^−6^(S)	0.0166(S)	2.14·10^−6^(S)	0.825	0.458
BPE-SP-PBS	roots	0.0103 (I)	0.234	0.352	0.513	0.822
	shoots	9.28·10^−5^(S)	2.19·10^−6^(S)	0.000194(S)	0.0214(S)	4.01·10^−6^ (I)
Tested compound	Exposed plant organ	*p*-values for SIA				
		Concentrations (% *w/w*)				
		0.02	0.095	0.48	2.38	11.9
BPE-AMF-PLA	roots	0.000545(I)	0.00654(I)	0.612	0.00626(I)	0.0812
	shoots	0.959	0.596	0.843	0.528	0.0142(I)
BPE-T-PHBV	roots	0.737	0.0196(I)	0.223	0.465	0.993
	shoots	0.0584	0.454	0.509	0.517	0.000444(I)
BPE-C-PLA	roots	0.375	0.969	0.599	0.791	0.677
	shoots	0.987	0.00235(S)	0.0133(S)	0.0632	0.979
BPE-RP-PLA	roots	0.394	0.116	0.349	0.000938(I)	0.0332(I)
	shoots	0.577	0.659	0.634	0.224	0.0955
BPE-SP-PBS	roots	0.0506	0.311	0.214	0.0416(I)	0.000111(I)
	shoots	0.00984(S)	0.0345(S)	0.329	0.214	0.110

(I)—inhibition; (S)—stimulation.

## Data Availability

The data presented in this study are available on request from the corresponding author.
